# Radiological and clinical examination in the diagnosis of Spigelian hernias

**DOI:** 10.1308/003588413X13511609957092

**Published:** 2013-03

**Authors:** D Light, D Chattopadhyay, S Bawa

**Affiliations:** Northumbria Healthcare NHS Foundation Trust, UK

**Keywords:** Spigelian hernia, Ultrasonography, Occult hernia

## Abstract

**Introduction:**

Spigelian hernia are rarely reported lateral abdominal wall hernias. Clinical diagnosis of a suspected hernia can be challenging owing to vague presenting symptoms and signs. This study aimed to investigate the accuracy of preoperative imaging and clinical examination in the diagnosis of Spigelian hernias.

**Methods:**

A retrospective analysis was performed of patients who presented to North Tyneside and Wansbeck General Hospitals between 1998 and 2010. All patients were assessed by a consultant general surgeon in the outpatient clinic or on the surgical admissions ward. Patients were included who presented with a history suggestive of a Spigelian hernia and a palpable lump or equivocal clinical examination. All patients proceeded to surgery, which was used as the reference standard.

**Results:**

Overall, correlation with operative findings showed computed tomography (CT) to have a sensitivity of 100% and a positive predictive value (PPV) of 100%. Ultrasonography had a sensitivity of 90% and a PPV of 100%. Clinical assessment alone had a sensitivity of 100% and a PPV of 36%.

**Conclusions:**

This study shows that ultrasonography and CT have a high sensitivity and PPV in relation to occult Spigelian hernias. When no obvious Spigelian hernia is present, patients should be evaluated with radiological investigation to establish a diagnosis. Owing to diagnostic uncertainty, a laparoscopic approach should be favoured.

Spigelian hernias are rarely reported lateral abdominal wall hernias. A Spigelian hernia arises from the Spigelian fascia, which lies along the semilunar line lateral to the rectus abdominis muscle. The external oblique aponeurosis covers the Spigelian fascia, which can make clinical assessment of a suspected hernia difficult.^1^

Patients may present with a palpable lump with the classical findings of a hernia. In this case, the diagnosis is relatively straightforward. However, a number of patients present with no obvious hernia but a vague lump or tenderness along the region of the Spigelian fascia. In these patients, it can be particularly difficult to diagnose a true Spigelian hernia.

A number of radiological investigations have been used to diagnose Spigelian hernias. Ultrasonography has been shown to offer an accurate evaluation of suspected Spigelian hernias in a number of case reports.^2–4^ It offers a non-invasive and dynamic assessment of soft tissues. It does have limitations in obese patients and is an operator dependent technique. It has been shown to have a variable accuracy in occult inguinal hernias.^5,6^

Computed tomography (CT) has also been shown to be of value in the diagnosis of Spigelian hernias.^7–9^ However, CT is associated with a radiation exposure, which limits its role as a first line imaging modality for hernia.

Herniography has also been evaluated in diagnosing Spigelian hernias and has been shown to be accurate as a diagnostic tool.^10^ In a case report comparing herniography with ultrasonography, a Spigelian hernia was not seen on ultrasonography but was confirmed on herniography.^11^ Herniography does have a number of disadvantages in that it involves a risk of visceral injury and allergic reaction to contrast medium.^12^ As a result, it has become less widely used in recent years.

Owing to the variability of results for imaging, some surgeons will proceed to a diagnostic laparoscopy directly to offer a true evaluation of the presence of a hernia. The aim of this study was to examine the role of radiological imaging combined with clinical judgement in detecting the presence of Spigelian hernias.

## Methods

A retrospective analysis was carried out of patients who presented to North Tyneside and Wansbeck General Hospitals between 1998 and 2010. Computer records and medical case notes were reviewed. Only patients 18 years and over were included in the study. All patients were assessed by a consultant general surgeon in the outpatient clinic or on the surgical admissions ward. Patients who had clinically palpable hernias were excluded from the study. Patients were included if they presented with a history suggestive of a Spigelian hernia but had a normal or equivocal clinical examination. All patients proceeded to surgery, which was used as the reference standard.

A group of patients was assessed preoperatively with ultrasonography, which was performed supine and erect together with dynamic manoeuvres to demonstrate a hernia. This was performed by an experienced consultant radiologist or radiographer. A separate group of patients was investigated preoperatively by helical CT. This was performed by a consultant radiologist.

These patients were reassessed in the outpatient clinic following radiological investigation and the decision was made to operate by a consultant general surgeon. Surgery was undertaken by either open or laparoscopic repair.

A further group of patients proceeded to diagnostic laparoscopy directly from initial presentation in the outpatient clinic without imaging studies. Operative records were then reviewed for the presence or absence of a hernia.

## Results

Data were analysed from 54 patients. The mean patient age was 62 years and 57% were female. Ultrasonography was performed by a consultant radiologist in 90% of cases. Thirty-three cases were performed laparoscopically and twenty-one as an open procedure.

There were two distinct groups in this study. In the first group, a firm clinical diagnosis was made based on history and a palpable lump on clinical examination. The second group did not have a clinically palpable lump and the patients either underwent preoperative imaging or proceeded directly to surgery based on clinical judgement.

There were 31 patients in the first group. All patients proceeded to surgery. At surgery, the diagnosis was confirmed in 30 patients and a repair was performed. In one case, a lipoma had been misdiagnosed as a hernia.

There were 23 patients in the second group. There was variability in the investigation and management of patients in this group owing to variation in individual clinicians’ practice. Three patients underwent CT, eleven underwent ultrasonography and nine proceeded directly to a laparoscopy from initial clinical presentation. The overall results for this group are presented in Table 1.

**Table 1:** Operative findings compared with radiological/clinical preoperative diagnosis

Investigation	True positive	False positive	False negative	True negative	Sensitivity	Positive predictive value
Computed tomography	3	0	0	0	100%	100%
Ultrasonography	9	0	1	1	90%	100%
Clinical suspicion alone	5	4	0	0	100%	36%

In the CT group, all three patients had a positive result confirmed by surgery. One patient required a small bowel resection for strangulation.

Of the eleven patients in the ultrasonography group, nine had positive ultrasonography. All these patients had a Spigelian hernia confirmed during surgery. Laparoscopic repair was undertaken in six patients and three patients had an open repair. Two patients proceeded to surgery despite a negative ultrasonography due to ongoing pain and a Spigelian hernia was found in one case during laparoscopy.

Nine patients with no palpable hernia proceeded directly to laparoscopy without imaging studies. A Spigelian hernia was found in five cases. No hernia was found in four patients.

Overall correlation with operative findings showed CT to have a sensitivity of 100% and a positive predictive value (PPV) of 100%. Ultrasonography had a sensitivity of 90% and a PPV of 100%. Clinical assessment overall had a sensitivity of 100% and a PPV of 87%. When there was no palpable hernia, clinical judgement alone had a sensitivity of 100% and a PPV of 36%.

## Discussion

The Spigelian hernia was first described by Henri Francois Le Dran in 1742 and named posthumously after Adriaan van den Spiegel, a Belgian anatomist of Padua. The Spigelian fascia extends bilaterally from the ninth rib to the pubic tubercle. Its lateral border is the fibres of the internal oblique muscle and its medial border is beneath the point where the external oblique aponeurosis becomes the anterior rectus sheath. Deep to the fascia lies preperitoneal fat and peritoneum. The aponeurosis of external oblique lies superficially. A hernia may occur anywhere along the Spigelian fascia although it is more common in the region where the semicircular line crosses the semilunar line.^1^ As a hernia is covered by the external oblique muscle, it may be difficult to feel the hernia on clinical examination.

As a result of such diagnostic uncertainty, there is a need for accurate imaging to establish a diagnosis. A number of modalities have been described. Ultrasonography and CT have become the most commonly used modalities in the evaluation of Spigelian hernias. Due to the possibility of false negative radiological examinations, some surgeons proceed directly to a laparoscopy as this remains the gold standard in the assessment of Spigelian hernias. However, this is associated with the risks of anaesthesia and complications of laparoscopic surgery.

This study shows ultrasonography and CT to offer an accurate evaluation for suspected Spigelian hernias. Over half of the patients who underwent surgery based on clinical examination alone had no hernia present. It could be said that clinical assessment alone is inaccurate as a diagnostic tool for occult Spigelian hernias. An important factor is that surgery itself is associated with risks and complications. This exposure can be minimised by accurate preoperative assessment and investigation. It is strongly recommended that clinically occult Spigelian hernias should be assessed with radiological investigations before proceeding to surgery.

A number of studies on Spigelian hernias have shown a high rate of incarceration and concurrent risk of strangulation.^13–15^ As a result, operative repair is recommended when a hernia is identified. There is an increasing trend for laparoscopic repair of Spigelian hernias.^16^ The safety of laparoscopic repair is well demonstrated with a low recurrence rate in a systematic review from 2004.^17^ Our study offers further justification of this technique as the diagnostic uncertainty related to Spigelian hernias means that a number of patients would proceed to surgery when there is no true hernia present. In such cases, laparoscopy offers a less invasive procedure with the subsequent postoperative benefits.

A study by Paajanen *et al* demonstrated an incidence of 21% for unexpected hernias during laparoscopy, with a 2% incidence of Spigelian hernias.^18^ A laparoscopic approach offers advantages in that the contralateral side may be assessed during surgery, granting the feasibility of bilateral repair if necessary. A laparoscopic approach also has advantages in that a number of cases have been described where an inguinal hernia tracking through the tissue plane between the external and internal oblique was misdiagnosed as a Spigelian hernia.^19^ This would present access difficulties in an open repair, which is not present in a laparoscopic approach.

This study evaluated patients assessed preoperatively and taken for surgery. It is not possible to give true values for sensitivity and specificity as not all patients who underwent investigation proceeded to surgery. A prospective multicentre study in which patients with symptoms suggestive of a Spigelian hernia but who had no palpable hernias were assessed would be valuable in evaluating this field further.

## Conclusions

This study shows that ultrasonography and CT have a high sensitivity and PPV in relation to occult Spigelian hernias. In this setting, clinical assessment alone is not accurate in making a decision to operate. When no obvious Spigelian hernia is present, patients should be evaluated with radiological investigation to establish a diagnosis. Owing to diagnostic uncertainty, a laparoscopic approach should be favoured.
